# Effect of Nutraceutical Factors on Hepatic Intermediary Metabolism in Wistar Rats with Induced Tendinopathy

**DOI:** 10.3390/ijms25010629

**Published:** 2024-01-03

**Authors:** Marta Ramos-Barbero, Eva E. Rufino-Palomares, Sergio Serrano-Carmona, Manuel Hernández-Yera, Leticia García-Salguero, José Antonio Lupiáñez, Amalia Pérez-Jiménez

**Affiliations:** 1 Department of Biochemistry and Molecular Biology I, Faculty of Science, University of Granada, 18071 Granada, Spain; ramosb@correo.ugr.es (M.R.-B.); elgarcia@ugr.es (L.G.-S.); jlcara@ugr.es (J.A.L.); 2 Sergio Serrano Fisiomedicina Avanzada, Physiotherapy Clinic, 41011 Sevilla, Spain; drserranocarmona@gmail.com; 3 Department of Zoology, Faculty of Science, University of Granada, 18071 Granada, Spain

**Keywords:** Achilles tendon, aspartate, glycine, hydroxytyrosol, maslinic acid, nutritional factors, rehabilitation, tendinitis

## Abstract

Tendinopathy (TP) is a complex clinical syndrome characterized by local inflammation, pain in the affected area, and loss of performance, preceded by tendon injury. The disease develops in three phases: Inflammatory phase, proliferative phase, and remodeling phase. There are currently no proven treatments for early reversal of this type of injury. However, the metabolic pathways of the transition metabolism, which are necessary for the proper functioning of the organism, are known. These metabolic pathways can be modified by a number of external factors, such as nutritional supplements. In this study, the modulatory effect of four dietary supplements, maslinic acid (MA), hydroxytyrosol (HT), glycine, and aspartate (AA), on hepatic intermediary metabolism was observed in Wistar rats with induced tendinopathy at different stages of the disease. Induced tendinopathy in rats produces alterations in the liver intermediary metabolism. Nutraceutical treatments modify the intermediary metabolism in the different phases of tendinopathy, so AA treatment produced a decrease in carbohydrate metabolism. In lipid metabolism, MA and AA caused a decrease in lipogenesis at the tendinopathy and increased fatty acid oxidation. In protein metabolism, MA treatment increased GDH and AST activity; HT decreased ALT activity; and the AA treatment does not cause any alteration. Use of nutritional supplements of diet could help to regulate the intermediary metabolism in the TP.

## 1. Introduction

The term tendinopathy (TP) defines the clinical syndrome characterized by a combination of pain, inflammation, and impaired tendon performance, usually due to its overuse, traumatism, or pathologies [1]. The etiology of this condition remains unclear, although hypoxia, ischemic damage, oxidative stress, hyperthermia, impaired apoptosis, inflammatory mediators, and matrix metalloproteinase (MMP) imbalance have been studied as possible causes [2]. In addition, age, gender, and genetics are considered risk factors [3]. These factors as genetic conditions contribute to both localized inflammation and degeneration of collagen fibers that have been implicated in the study of TP. The affected genes are either related to abnormal collagen production levels or collagen malformation [4].

Epidemiological studies of TP focus on two main factors, extrinsic and intrinsic. As extrinsic factors can be included the overuse of tendons due to physical activity and environmental conditions as risk factors, including the use of drugs. The intrinsic factors are associated with metabolic, systemic, neurological, and infectious diseases. Moreover, they can also include renal failure, psoriasis, hyperparathyroidism, and hyperthyroidism [5]. Histologically, TP is characterized by the absence of inflammatory cells, poor healing, intratendinous degeneration, disorientation and thinning of collagen fibers, hypercellularity with high concentrations of glycosaminoglycans and proteoglycans, and neovascularization [6].

TP consists of three phases that overlap in time. The first phase is the acute inflammatory phase, in which erythrocytes and inflammatory cells rush to the affected area [7]. Erythrocytes and inflammatory cells migrate to the site of injury in the first hours after injury. Vascular permeability increases, angiogenesis, tenocyte proliferation, and collagen fiber production are initiated by the release of vasoactive and chemotactic factors [8]. In the second phase, named the proliferation phase, collagen associates with fibroblasts to form immature fibrils, which subsequently attach to the end of other fibrils to grow linearly [9]. In this second stage, type III collagen synthesis peaks and lasts for a few weeks. In addition, water content and glycosaminoglycan concentrations remain high during this stage [8]. The last one is the remodeling phase, in which collagen fibers increase and improve the strength, elasticity, and structure of the tendon [7]. Fibroblasts, with their oval core, interpose themselves between the collagen fibers they produce and, progressively, their core elongates [9]. The repaired tissue changes from cellular to fibrous and the collagen fibers align in the direction of the loads applied to the tendon [8].

TPs have no effective treatment to date, with invasive procedures having the most negative side effects. However, treatment of TP with non-invasive nutritional factors, or in combination with other existing therapeutic approaches, could open up a new line of research. Since TP is characterized by irregular or disturbed homeostasis, poor nutrition can lead to the development of this disease [3]. However, adequate food intake of both macro- and micronutrients may be a plausible strategy in the prevention and amelioration of the disease, with potential benefits of nutrition on tendon health emerging [10]. It is necessary to understand the effect of the interaction of these substances with cell and tissue biology and, thus, use nutritional management as a unique tool towards the treatment of TP [11,12].

Functional foods and nutraceuticals, including natural compounds derived from the oleic industry, such as hydroxytyrosol and maslinic acid, have been shown to possess numerous bioactive properties, including anti-inflammatory, antioxidant, anticarcinogenic, and other properties [13,14]. In addition, it is well known that the amino acids glycine and L-aspartic acid play an essential role in collagen synthesis [15].

Hydroxytyrosol (3,4-dihydroxyphenyl ethanol; HT) is one of the most potent antioxidants known. HT has been shown to prevent cytokine formation, nitric oxide (NO) generation, tumor necrosis factor-alpha (TNF-α) secretion, and mRNA expression and to inhibit the expression of nitric oxide synthase (iNOS) and cyclooxygenase (COX-2). Some research has shown that HT treatment prevents acanthocyte formation observed after HgCl_2_ exposure and prevents HgCl_2_ induced oxidative stress damage, including ROS production, lipid peroxidation, and oxidation of the sulfhydryl group of total proteins in the plasma membrane [16].

Maslinic acid (2-α, 3-β-dihydroxyolean-12-en-28-oic acid; MA) is a triterpenoid derived from plants, such as olive, which prevents the generation of proinflammatory cytokines and oxidative stress. MA, in addition to its anti-inflammatory properties, has anticarcinogenic, antidiabetic, antimicrobial, neuroprotective, and hepatoprotective properties [17].

Glycine (Gly) is an amino acid that modulates the systemic inflammation cascade and inhibits TNF-α and IL-1β. Previous studies have demonstrated the beneficial effects of a glycine diet on the remodeling process of inflamed tendons following tendinopathy. A glycine-rich diet has benefits on biochemistry, structure, and biomechanics in the Achilles tendon with TP due to its involvement in the molecular structure of collagen [18]. Aspartic acid (Asp) is important for its role in matrix synthesis and degradation. In tendinopathies, there is an imbalance of matrix metalloproteases, which leads to a failure in the remodeling of the lesions. Asp must be incorporated in its L-form into proteins, otherwise, if it is not, or if its D-form is increased, there is a slower matrix turnover, leading to problems of matrix synthesis and tendon physiology, with the mechanical effects that this entails [19]. A mix of Gly and Asp was used in this study (AA).

All these bioactive molecules (HT, MA, and AA) could support tendon regeneration, as well as the prevention of chronic tendinopathies.

The aim of this study is to understand and analyze biomolecular processes by studying the modulatory effects of HT, MA, and AA on hepatic intermediary metabolism and plasma metabolites produced in the healthy and pathological Achilles tendon during the different phases of the pathology.

## 2. Results

### 2.1. Interaction between Parameters

The results showed interaction between the factors analyzed. The effects of the different treatments on all the parameters studied were analyzed using two-way analysis of variance (two-way ANOVA), as shown in Table 1.

### 2.2. Growth Trial

All animals were subjected to weight monitoring throughout the experiment. The growth performance mean weight variations according to the experimental group are shown in Table 2.

### 2.3. Activity of Intermediary Metabolism Key Enzymes

The enzymatic activity of the main enzymes involved in intermediary metabolism was modified in response to different treatments assayed and in the different phases of tendinopathy. The results for each of the enzymes assayed in each of the phases are shown below in Table 3 (Phase I), Table 4 (Phase I–II), Table 5 (Phase II), and Table 6 (Phase III).

T-HK activity was significantly modified when comparing between TP phases, but not between treatments. Thus, the control group (C) significantly increased its T-HK activity in interphase (Phase I–II), then decreased in the proliferation phase (Phase II) and significantly increased in the remodeling phase (Phase III). The DC and HT groups showed no change in the activity of this enzyme throughout the disease. However, the MA treatment showed a reduction in T-HK activity in the transition phase of TP (Phase I–II) and an increase in the last phase (Phase III). Finally, the AA group showed an increase in T-HK activity in Phase I–II compared to Phase I, which was then reduced in Phase II and increased in Phase III (*p* < 0.05) (Table 3, Table 4, Table 5 and Table 6). Results between treatments within the same phase on T-HK showed significant differences between MA and AA (Gly + Asp) groups with respect to the DC group only in the proliferation phase (Phase II). Similar T-HK activities were found in MA and C conditions and AA was the treatment that had the lowest T-HK activity found in Phase II (*p* < 0.05) (Table 5).

PK activity showed changes when compared to phases of TP but not between treatments. Thus, the DC and MA groups were the only ones to be significantly modified. The DC group showed a reduction of PK in the transition phase (Phase I–II) with respect to the inflammatory phase (Phase I) and significantly increased its activity in Phase II and III, with Phase III showing the highest activity. The MA group showed an increase in activity in the remodeling phase (Phase III) with respect to the proliferation phase (Phase II) (*p* < 0.05) (Table 3, Table 4, Table 5 and Table 6). In addition, PK showed significant differences between treatments, within phases. In Phase I, PK activity was significantly decreased in MA and AA groups with respect to DC (Table 3). However, the transition phase (Phase I–II) showed no significant differences between treated groups and controls (Table 4). In Phase II, a significant decrease in PK activity was observed in the MA compared to the DC group (*p* < 0.05) (Table 5). In the last phase of the TP (Phase III), a decrease in PK activity was noted in the AA compared to the DC group (*p* < 0.05) (Table 6).

FBPase activity was not altered in either the healthy (C) or diseased control (DC) groups with respect to the different phases of TP (*p* < 0.05) (Table 3, Table 4, Table 5 and Table 6). In contrast, all groups treated with nutritional factors showed changes in the FBPase activity in the course of the disease. The HT group increased its FBPase activity in Phase III of the disease. The MA group significantly increased activity in Phase I–II, decreased it in Phase II, and increased it again in Phase III. Finally, the AA group showed a significant increase in FBPase activity in Phase III (*p* < 0.05) (Table 3, Table 4, Table 5 and Table 6). When comparing the FBPase enzyme activity between the different phases of TP, results showed that in Phase I, FBPase activity was significantly lower in the MA group compared to DC (Table 3). In Phase I–II and Phase III, neither group treated with nutritional factors differed significantly from the DC group (Table 4 and Table 6). In Phase II, the MA and AA groups had significantly lower FBPase values than the DC group (*p* < 0.05) (Table 5).

The activity of the enzyme G6PDH was altered when comparing the different phases of TP. The healthy control group (C) showed a significant reduction of G6PDH activity in the remodeling phase (Phase III, Table 6). The diseased control group (DC) decreased this enzyme activity in the transition phase (Phase I–II, Table 4) with respect to the inflammation phase (Phase I). The HT group showed increased activity in Phase III compared to Phase I (Table 6). The MA group did not induce significant changes in G6PDH activity in any of the phases. Finally, the AA group showed a decrease in G6PDH activity in Phase III with respect to the two previous phases (Phase I–II, II) (*p* < 0.05) (Table 4, Table 5 and Table 6). In Phase I, HT, MA, and AA treatments showed a lower G6PDH activity when comparing the DC and C groups (*p* < 0.05, Table 3) and no significant differences between treatments were observed in Phase I–II and II of TP (Table 4 and Table 5). However, in Phase III the G6PDH levels in the HT and MA groups were significantly higher than in the DC group (*p* < 0.05) (Table 3, Table 4, Table 5 and Table 6).

With regard to LDH activity, significant differences were observed between the different phases in each treatment, except in the group treated with MA. In the C group, differences were observed between Phase II (where LDH activity is lower) and the other phases of tendinopathy (*p* < 0.05) (Table 5). In the DC group, lower LDH activity was observed in Phase II compared to Phases I–II and III (*p* < 0.05) (Table 4, Table 5 and Table 6). In the HT condition, significant differences were found between Phases I, II, and III, with higher LDH activity in Phase III and lower LDH activity in Phase II (*p* < 0.05) (Table 3, Table 4, Table 5 and Table 6). The AA group showed a significant decrease between Phase II and Phases I, I–II, and III (*p* < 0.05) (Table 3, Table 4, Table 5 and Table 6). There were no significant differences in LDH activity in the different phases between the nutritional treatments (HT, MA, and AA).

CS activity showed changes between phases of tendinopathy in MA and AA groups but did not show variations in the other groups. The MA group presented a significant increase in CS activity in the transition phase (Phase I–II) with respect to the inflammatory phase (Phase I) to return to similar levels in the remodeling phase (Phase III) (*p* < 0.05) (Table 3, Table 4, Table 5 and Table 6). However, the AA group showed significantly lower levels in Phases II and III compared to Phase I–II (*p* < 0.05) (Table 4, Table 5 and Table 6). CS activity showed no differences between treatments within Phases I and III (*p* < 0.05) (Table 3 and Table 6). There were also no significant differences in Phased I–II between HT, MA, and AA and C and DC groups, while C and DC groups were different (*p* < 0.05) (Table 4). In Phase II, the AA group showed significantly lower CS activity compared with C and DC treatments (*p* < 0.05) (Table 5).

With respect to lipid metabolism (FAS, EM, and HOAD activities), there were observed differences in treatments and phases (Table 3, Table 4, Table 5 and Table 6). In group C, FAS activity is decreased in Phase I, increasing in Phase I–II, until it reaches a significant maximum in Phase II (*p* < 0.05) (Table 3, Table 4 and Table 5). The MA group presented significantly higher levels in Phase I–II compared to all other phases (Table 4). The AA group showed a progressive decrease in activity during the course of the phases (*p* < 0.05) (Table 3, Table 4, Table 5 and Table 6). In Phase I, the HT group had significantly higher levels of FAS activity than the control groups (C and DC); the MA group had significantly lower activity than the DC group; and the AA group had significantly higher levels than the C group but not the DC group (*p* < 0.05) (Table 3). In Phase I–II, the AA group showed significantly lower levels of FAS activity than the DC group (*p* < 0.05) (Table 4). Phase II was characterized by an increase in FAS activity in group C compared to all other groups (*p* < 0.05) (Table 5). Finally, in Phase III, there was a significant decrease in FAS activity in the MA group with respect to DC (*p* < 0.05) (Table 6).

ME activity showed significant differences between disease phases in C, HT, AM, and AA groups. The C group showed a significant increase in ME activity in the transition phase (Phase I–II) and a significant decrease in the proliferation phase (Phase II) (*p* < 0.05) (Table 4 and Table 5). In the HT group, significant reduction in ME activity during the first three phases was found (Phases I, I–II, and II) (*p* < 0.05) (Table 3, Table 4 and Table 5). The MA group showed a significant increase in activity in Phases I–II and III with respect to the other phases (Table 3, Table 4, Table 5 and Table 6). Lastly, the AA group presented maximum levels of this enzyme in Phase I–II (*p* < 0.05) (Table 4). With regard to the differences between treatments within each phase, it is noteworthy that in the inflammation phase (Phase I), the MA and AA groups showed lower levels of ME activity than the DC group (*p* < 0.05) (Table 3). In Phase II, the AA group showed lower levels of ME activity than the DC group (Table 5). However, in Phase III, the ME activity in HT, MA, and AA groups did not differ with respect to the DC group, although the HT and MA groups exhibited higher levels of ME activity than the C group (*p* < 0.05) (Table 6).

HT and DC groups did not present significant differences between phases of TP in the HOAD activity. The C group showed an increase in HOAD activity in the Phases I–II, II, and III with respect to Phase I, with the highest activity being found in Phase II (Table 5). The MA group showed significantly higher enzyme activity in Phase I–II compared to the other phases (Table 4). The AA group showed a maximum of HOAD activity in Phase III of the disease (*p* < 0.05) (Table 6). In Phase I–II, there was an increase in HOAD activity in the MA group with respect to the controls (C and DC) and a decrease in activity in the MA and AA groups with respect to the controls in Phase II (*p* < 0.05) (Table 4 and Table 5).

GDH, AST, and ALT activities were measured as key enzymes to protein metabolism. These enzymes were altered in the phases of TP and in response to the different treatments. GDH activity showed significant differences between phases only in the C and MA groups. The C group displayed a maximum activity of GDH in the proliferative phase of the disease (Phase II), while the MA group showed its maximum activity in the transition phase (Phase I–II) (*p* < 0.05) (Table 4 and Table 5). Furthermore, this GDH activity did not show variations between treatments (HT, MA, and AA) in the inflammatory phase (Phase I) and in the remodeling phase (Phase III). In the transition phase (Phase I–II), there was a significant increase in GDH activity in the MA group with respect to the C group but not with respect to the DC group (Table 4). Phase II showed a decrease in GDH activity of all treatments with respect to group C but not to DC (*p* < 0.05) (Table 5).

AST activity manifested in the MA treatment an increase in Phases I and I–II (Table 3 and Table 4), decreasing significantly during Phase II (Table 5) to increase again in Phase III (Table 6). In the AA group, significant differences were only observed between Phases II and III, with minimum and maximum AST activity values, respectively (*p* < 0.05) (Table 5 and Table 6). Regarding the differences between treatments within the same phase of TP, an increase in AST activity was observed in the MA group in Phase I–II (Table 4) and a significant decrease in activity in the MA and AA groups with respect to the C group in Phase II (*p* < 0.05) (Table 5).

Finally, ALT activity showed differences between phases of TP only in the HT and MA groups. Both groups showed a significant increase in ALT activity in Phases I–II and III, with this increase being higher in the latter phase (Phase III) (Table 4 and Table 6). Focusing on the differences between treatments within the same phase of TP, a significant decrease in activity was observed in the HT group with respect to the DC group in Phase I and a significantly higher increase in the AA group with respect to the C group (Table 3). In Phase II, a significant decrease of ALT activity was observed in the HT and MA groups with respect to the DC group (Table 5). In Phase III, an increase occurred in ALT activity in all treatment groups (HT, AM, and AA) with respect to the control groups (C and DC) (*p* < 0.05) (Table 6).

## 3. Discussion

Tendinopathy (TP) is a chronic disease with difficult therapeutic problems for the patient as well as the health care professional because the etiology and management are uncertain. Although different treatment strategies have been studied against TP, the current ones are not completely effective because they do not definitively resolve the disease. In recent decades, several non-surgical treatment modalities have been introduced, with an increasingly relevant role of local drug injections, such as sclerosing agents, corticosteroids, and high-volume image-guided injections and physiotherapy ranging from external shock waves to electric fields and intratissue transcutaneous electrolysis [11,20]. Non-steroidal anti-inflammatory drugs (NSAIDs) and steroidal anti-inflammatory drug treatments produce loss of fibrous tissue, cell death, and loss of mechanical strength of muscles [21]. All these treatments tested against TP lack a solid scientific basis [20]. This is why the search for new treatments is necessary in order to combat this pathology. The aim of this study was to determine the possible modulatory effects of three or several potential nutraceutical compounds, namely hydroxytyrosol (HT), maslinic acid (MA), and a mix of glycine and aspartic acid (AA), on the activity of key enzymes of intermediary metabolism in the liver of Wistar rats with induced TP throughout the different phases of the disease. To this end, the possible modifications of glucose, lipid, and protein metabolism on T-HK, PK, FBP, G6PDH, LDH, CS, FAS, ME, HOAD, GDH, AST, and ALT enzymes were evaluated during the development of the different stages of TP.

It is well known that nutrition has an impact on human health. Nutritional components can be a good ally in the prevention of diseases because they possess important biological properties such as anticancer, antioxidant, anti-inflammatory, and antimicrobial properties, among others [22]. Natural compounds such as HT and MA have not yet been studied as anti-inflammatories against TP, but it would be important since this bioactivity improves the lipid profile, reducing oxidative stress and activating inflammatory cells [23]. This is the first evidence of these compounds (HT and MA) and AA as possible treatments for this pathology (TP).

Recent findings have demonstrated that glucose metabolism is disturbed during injury and recovery in TP in both humans and mice [10]. These changes include increases in glucose, lactate, and pyruvate contents in healing human Achilles tendons [10] and stimulation of glycolysis, lactate synthesis, and TCA cycle in injured mouse Achilles tendons [11]. The lower T-HK activity found in the MA and AA groups in Phase II may be because the function of T-HK is to phosphorylate glucose using ATP to form glucose-6-phosphate [24]. An increase in these enzymes in the proliferation phase in the DC group indicates a down-regulation of glucose levels. Previous studies have shown how tendon inflammation may be due to reduced response to damage and how a high-glucose environment can lead to chronic tissue inflammation and, therefore, the untreated group has higher levels of enzymes involved in glucose metabolism [25].

One biological activity shown by MA is related to the modulation of glycogen metabolism, inhibiting glycogen phosphorylase [26]. This fact explains why MA treatment lowers PK activity in Phases I and II. On the other hand, the amino acid Gly is involved in biological functions such as protein synthesis and collagen production. This function of Gly implies that it plays important roles in cellular metabolism and thus may influence specific metabolic pathways, such as glycolysis [27]. It is noteworthy that glycolysis may influence inflammatory response [28] and the PK activity modulates glycolysis, therefore, the lower activity of PK in nutritional factors and amino acid groups may indicate an early anti-inflammatory effect, which does not occur in the untreated diseased (DC) group.

The function of FBPase is to participate in gluconeogenesis, which can be influenced by inflammation leading to metabolic stress. Therefore, when there is a prolonged presence of inflammation, as in the case of the untreated (DC) group, gluconeogenic processes will be activated to provide a continuous source of glucose [29]. The DC group has higher FBPase activity due to the demand for glucose to repair the damage caused by PT. Regarding MA and AA in Phase II, the disease groups have lower FBPase activity levels due to earlier regeneration of the injury, and therefore less inflammation. Glycogen can play an important role during inflammation by providing a source of quick energy and contributing to metabolic regulation. In situations of acute inflammation, such as infection or injury, the body often experiences increased metabolic stress. Under these conditions, mobilization of stored glucose in the form of glycogen may be essential to meet the energy demands of the immune system and other affected tissues [30].

On the other hand, the enzyme G6PDH plays a vital role in preventing cell damage by reactive oxygen species [31] and therefore may be elevated by the acute inflammation of the first phase (Phase I) of TP, in which there is a migration of proinflammatory molecules to the site of injury [32]. However, when treatment is effective, the activity of this enzyme should be reduced because its presence is not required since inflammation will have disappeared. Thus, in the absence of treatment, normal tendon function has not been restored and the tendon is still injured and the inflammation continues. This fact explains why the DC group, which does not receive any treatment, has higher levels of G6PDH activity in the late phase (Phase III) of the disease. However, the group treated with AA, which are involved in the formation of collagen synthesis, was able to regenerate the tendon more rapidly, and therefore reduce the presence of enzymes associated with damage [33]. Some authors have observed that type I collagen in TP mice increases after administration of Gly, improving the biomechanical properties of the tendon and the degree of fiber compaction [18]. This result is in concordance with results obtained in this study of the AA group regarding G6PDH activity.

LDH activity showed significant changes between the different phases of TP in each treatment, with the exception of the MA group. These results are consistent with other studies that measured LDH isoenzymes in patients with healthy and damaged tendons. The specific LDH activity of hypokinetic tendons was 13–66% of the specific activity of normal tendons [34]. A possible explanation for this phenomenon is that tendon damage causes an interruption of movement and, therefore, an alteration of tendon metabolism, reducing LDH activity in the different phases of TP where tendon recomposition predominates, i.e., a reduction of enzyme activity especially in Phase II or the proliferation phase, where all tendon repair processes are active, regenerating new fibers [34].

The enzyme citrate synthase (CS) acts in the first step of the Krebs cycle and is commonly used as a quantitative enzyme marker for the presence of intact mitochondria [35]. Although there is no evidence of a direct connection between AA and CS, the reduction in activity in the proliferation phase (Phase II) with respect to the DC group may be due to the involvement of amino acids in protein synthesis and their influence on complex metabolic pathways. So, glycine may lead to a reduction in inflammation in early stages of the disease through inhibition of proinflammatory cytokines [36].

FAS enzyme is involved in numerous biological processes related to lipid metabolism. It actively participates in the synthesis of membrane components necessary for cell division, protein modification, cell signaling, and proliferation [37] and redistributes the energy generated for protein synthesis [38]. The results found in this study showed an increase in FAS activity in the diseased control (DC) group, indicative of the need for cell synthesis and proliferation until late phases of the TP. This higher synthesis and proliferation are related to a reduction of FAS activity in MA and AA groups, from the early phases to the late phases of the TP, indicating an early tendon recovery when the injury is treated with these nutritional factors and amino acids.

ME activity was lowest in the AA group with respect to the DC group only in Phase I. This fact added to the subsequent rise in the final phase of TP may be due to the disease’s effect on oxidative stress, inducing metabolic disorders, as has been described in previous studies in liver injury, in which the authors concluded that ME enzyme did not participate in the regenerative processes of the tissue and therefore did not need to increase its expression levels for greater recovery [39]. The greater activity of ME found in the MA group supplies NADPH molecules to the liver which are key for the antioxidant response in which MA is implicated [22]. This result is also supported by the higher G6PDH activity found in the MA group.

HOAD activity showed an increase in the MA group with respect to the DC in the transition phase (Phase I–II) and a decrease in activity of the MA and AA groups with respect to the controls in Phase II. This effect can be explained by the relationship of amino acids in obtaining energy from the beta-oxidation of fatty acids. The group treated with AA could be provided with these compounds for early energy production and greater tendon recovery thanks to the decomposition of glycine and aspartic acid to enter these metabolic pathways and thus adapt to energy needs [33].

Proteins, along with lipids, are the main sources of energy. GDH plays an important role in the metabolism of amino acids, properly channeling the main nitrogen pathway of these compounds [22]. A significant decrease was only observed in the second phase of tendinopathy for the GDH enzyme in all treatments with respect to group C, probably due to its allosteric inhibition by the higher amount of NADPH and ATP produced for fiber repair [40].

The main tissue involved in protein catabolism is the liver, where the first step consists of the elimination of the amino group, which is catalyzed by transaminases, among others [22]. AST data show an increase in the activity in the MA group in Phase I–II and a significant decrease in activity in the MA and AA groups compared to the C group in Phase II. It has been observed that the levels of this enzyme increase when tissue damage or inflammation occurs, an event that has been demonstrated in other diseases and that would explain why anti-inflammatory factors such as MA and AA cause a decrease in this enzyme in the proliferation phase of TP [41]. Similarly, ALT activity shows significantly lower levels in the HT and MA groups compared to the DC group in Phase II. Additionally, in Phase III, an increase in ALT activity occurs in all treatment groups (HT, MA, and AA) with respect to the control groups. This confirms the presence of ALT activity in inflammatory processes, as group C, which did not have tendinopathy induced, had the lowest levels of activity [41]. HT and MA showed a significant increase in ALT activity in Phases I–II and III. This may be due to the fact that the remodeling phase is a period of intense activity in which mature tendon fibers are formed and recover elasticity and strength so that the tissue can regain its normal function [7].

The effect of HT in carbohydrate metabolism produced an increase in FBPase and decrease in G6PDH activity in Phase III compared to the control group. The decrease in the G6PDH activity could be explained by the fast recovery of TP with disappearing inflammation and the higher FBPase activity found is related to cell damage by reactive oxygen species [29,30]. In lipid metabolism, FAS levels in HT conditions showed a significant increase in the first phase of TP and a reduction of this activity in all other phases. This result suggests that the induced TP interferes with the effect of HT on lipogenesis. This same result has been observed previously, as HT can preserve the glycemic index, reduce triglyceride levels, and prevent inflammation and LDL oxidation [36,42]. In protein metabolism, a decrease in ALT activity was observed in the inflammation phase with respect to DC, while in the latter phase this activity is significantly higher in the HT group. This may be due to the fact that the remodeling phase is a period of intense activity in which mature tendon fibers are formed and recover elasticity and strength so that the tissue can revert to its normal function [7].

In carbohydrate metabolism, the lower T-HK, PK, and FBPase activities and higher G6PDH activity found in the MA treatment may be due to the fact that MA promotes glycogen uptake [43]. In lipid metabolism, MA produced less FAS activity and higher ME and HOAD activities. This decrease in FAS may be due to inhibition of this enzyme in a situation of inflammation, as observed in previous studies [22]. The increase in ME and HOAD activities in this experimental condition may because MA in lipolysis increased beta-oxidation during the first two phases of the disease, indicating a faster production of energy to be used in tissue regeneration [44]. In protein metabolism, MA produced a decrease in AST and ALT activities causing an increase in glutamate and aspartate to be incorporated into the new fibers that are synthesized during injury repair [38].

Finally, with respect to the carbohydrate metabolism in the AA group, lower T-HK, PK, FBPase, G6PDH, and CS activities were found. This is because the amino acids are used for the synthesis of new fibers during fibroblast repair [37], which would prevent aspartate from increasing glycolysis [45]. Moreover, these results are in concordance with an increase in the energy requirements during the remodeling phase of TP [38] and could indicate that TP modulates the effect of aspartate on increasing CS expression [46]. In lipid metabolism, the AA group produced a decrease in FAS, ME, and HOAD activities. This could imply that glycine and aspartate inhibit this metabolic pathway and this energy could be used for injury repair and tendinopathy recovery [38]. In protein metabolism, the higher ALT activity found in the AA group may be due to the fact that the remodeling phase is a period of intense activity in which mature tendon fibers are formed and recover elasticity and strength so that the tissue can regain its normal function [7].

## 4. Materials and Methods

### 4.1. Animals and Experimental Conditions

The present study was approved by the Ethics Committee of the University of Granada (authorization number 05/07/216/231). The animals were maintained at the facilities of the Institute of Biomedical Sciences (CIBM—University of Granada) during the experimental phase. A total of 64 male albino Wistar rats, Crl:CD (SD) code (Charles Rives, Laboratories International, Inc., Wilmington, MA, USA), with a mean initial body weight of 319.12 ± 0.89 g were homogeneously distributed in 5 triplicate groups: Healthy control (HC), diseased control (DC), hydroxytyrosol (HT), maslinic acid (MA), and amino acids (Gly + Asp). Rats were housed in plastic cages under controlled environmental conditions (12 h light/dark cycle) with free access to water and different experimental diets. Experimental diets were manufactured by ENVIGO RMS Spain S.L. The composition of the base diet in dry weight was 14.5% crude protein, 4.0% crude lipids, 4.5% crude fiber, and 4.7% ash. Vitamins and minerals were added to cover nutritional needs of the animals. This base diet was supplemented with 3 g of HT per kg diet, 0.65 g of MA per kg diet, and 28.1 + 9.4 g Gly + Asp per kg diet for the different experimental groups (HT, MA, and Gly + Asp). The composition of the diets is the same in terms of maslinic acid and hydroxytyrosol but differs in the diet with glycine and aspartate, with crude protein constituting 18.00% and crude ash 4%. To determine the approximate composition of the experimental diets (Table 7), AOAC methods were used (AOAC^®^ OFFICIAL METHODS) [47]. The maslinic acid (AM60^®^) and hydroxytyrosol (Olivesan^®^) were supplied by Extractos y Derivados S.L. (Granada, Spain). The glycine and the aspartate were purchased from Quimipur S.L.U. (Madrid, Spain).

The experiment lasted 41 days. On the first day, except for the HC group, tendinopathy was induced in the right Achilles tendon by administration of 50 µg of collagenase type I (Sigma-Aldrich, St. Louis, MO, USA) per 300 g of body weight. During the following 40 days, all groups were sampled at different times, coinciding with the phases of the tendinopathy process. Four samples were taken on days 6 (inflammatory phase), 13 (transition phase between inflammatory and proliferative), 26 (proliferative phase), and 40 (remodeling phase), coinciding with the different phases of tendinopathy. For sampling, the rats were anesthetized using sodium pentobarbital, from the commercial company Eutanax^®^, which was administered intraperitoneally at a dose that depended on the weight of the animal. When the state of sedation of the animal was verified, a needle was introduced into the left costal area of the animal from where 3 to 5 mL of blood was extracted from the heart with syringes that were previously heparinized. Then, by action of an overdose of sodium pentobarbital, the animals were sacrificed and the liver was extracted and immediately frozen in liquid nitrogen for subsequent biochemical analysis.

The extracted blood was centrifuged with a centrifuge model Sigma GmbH- 3K30 (Sigma Laborzentrifugen GmbH, Osterode am Harz, Germany) at 1000× *g* at 4 °C for 10 min in order to isolate the plasma from the cellular fraction. Liver was mechanically homogenized with a lysis buffer of 100 mM Tris HCl, 0.1 mM EDTA, and 0.1% Triton X-100, pH 7.8 at proportion 1/5 (weight/volume). Subsequently, the homogenate of each extract was centrifuged in an ultracentrifuge for 30 min at 30,000× *g*, at 4 °C. All samples were aliquoted and stored at −80 °C.

### 4.2. Quantification of Plasma Metabolites, Ions

Commercial kits were used for the determination of plasma metabolites: Glucose (Spinreact, Ref. 41010), triglycerides (Spinreact, Ref. 41031), total cholesterol (Spinreact, Ref. 41019), LDL cholesterol (Spinreact, Ref. MD41023), HDL cholesterol (Spinreact, Ref. MI1001096), and total lipid (Spinreact, Ref. 1001270) in the plasma. The concentration of total amino acids and soluble protein present in the plasma was determined according to Spies (1957) [48] and Bradford (1976) [49], respectively.

### 4.3. Intermediary Metabolism Enzyme Analysis

The analysis of the activity of each of the enzymes measured was performed by determining the variation of the optical density (OD) at 37 °C in a Synergy HTX plate reader and using the Gen 5™ software (version 2.0.0).

All enzymes were determined following the methods described by Pérez-Jiménez et al. (2009) [50], except FAS [51] and LDH.

#### 4.3.1. Hexokinase and Glucokinase

Briefly, hexokinase (HK; EC 2.7.1.1) and glucokinase (HK-IV; EC 2.7.1.2) activities were measured in a reaction mixture containing 50 mM imidazole-HCl buffer (pH 7.4), 2.5 mM ATP, 5 mM MgCl_2_, 0.4 mM NADP, 2 units mL^−1^ G6PDH, and 1 mM (HK) or 100 mM (HK-IV) glucose [50].

#### 4.3.2. Pyruvate Kinase

Pyruvate kinase (PK; EC 2.7.1.40) activity was measured in a reaction mixture consisting of 50 mM imidazole-HCl buffer (pH 7.4), 5 mM MgCl_2_, 100 mM KCl, 0.15 mM NADH, 1 mM ADP, 2 units mL^−1^ LDH, and 2 mM PEP [50].

#### 4.3.3. Fructose 1,6-Bisphosphatase

Fructose 1,6-bisphosphatase (FBPase; EC 3.1.3.11) activity was measured in a reaction mixture consisting of 50 mM imidazole-HCl buffer (pH 7.4), 5 mM MgCl_2_, 12 mM 2-mercaptoethanol, 0.5 mM NADP, 2 units mL^−1^ G6PDH, 2 units mL-1 PGI, and 0.5 mM fructose 1,6-bisphosphate [50].

#### 4.3.4. Glutamate Dehydrogenase

Glutamate dehydrogenase (GDH; EC 1.4.1.2) activity was measured in a reaction mixture containing 50 mM imidazole-HCl buffer (pH 7.4), 0.2 mM NADH, 1 mM ADP, 100 mM ammonium acetate, 2 units mL^−1^ LDH, and 10 mM α-ketoglutarate [50].

#### 4.3.5. Aspartate Aminotransferase

Aspartate aminotransferase (AspAT; EC 2.6.1.1) activity was determined in a reaction mixture containing 50 mM imidazole-HCl buffer (pH 7.4), 10 mM α-ketoglutarate, 0.3 mM NADH, 0.05 mM pyridoxal phosphate, 3 units mL^−1^ MDH, and 25 mM L-aspartate [50].

#### 4.3.6. Alanine Aminotransferase

Alanine aminotransferase (AlaAT; EC 2.6.1.2) activity was determined via a reaction mixture containing 50 mM imidazole-HCl buffer (pH 7.4), 10 mM α-ketoglutarate, 0.2 mM NADH, 0.05 mM pyridoxal phosphate, 2 units mL^−1^ LDH, and 25 mM L-alanine [50].

#### 4.3.7. Citrate Synthase

Citrate synthase (CS; EC 4.1.3.7) activity was measured in a reaction mixture containing 50 mM imidazole-HCl buffer (pH 8), 0.1 mM DTNB, 0.2 mM acetyl-CoA, and 0.2 mM oxalacetic acid [50].

#### 4.3.8. Glucose 6-Phosphate Dehydrogenase

Glucose 6-phosphate dehydrogenase (G6PDH; EC 1.1.1.49) activity was measured using a reaction mixture containing 50 mM imidazole-HCl buffer (pH 7.4), 5 mM MgCl_2_, 2 mM NADP, and 1 mM glucose-6-phosphate [50].

#### 4.3.9. Malic Enzyme

Malic enzyme (ME; EC 1.1.1.40) activity was measured in a reaction mixture containing 50 mM imidazole-HCl buffer (pH 7.4), 5 mM MgCl_2_, 0.4 mM NADP, and 2 mM L-malate [50].

#### 4.3.10. β-Hydroxyacyl-CoA Dehydrogenase

β-Hydroxyacyl-CoA dehydrogenase (HOAD; EC 1.1.1.35) activity was measured using a reaction mixture containing 50 mM imidazole-HCl buffer (pH 8), 0.1 mM NADH, and 0.1 mM acetoacetyl-CoA [50].

#### 4.3.11. Fatty Acid Synthase

Fatty acid synthase (FAS; EC 2.3.1.85) activity was measured in a reaction mixture containing 0.1 M potassium dihydrogen phosphate, 0.1 M dipotassium hydrogen phosphate trihydrate, 0.1 mM NADPH, 0.25 µM acetyl-CoA, and 100 mM phosphate buffer at pH 6.5 [51].

#### 4.3.12. Lactate Dehydrogenase

Lactate dehydrogenase (LDH; EC 1.1.1.27) was determined using the Spinreact quantitative LDH determination kit (ref: 41222), following the manufacturer’s specifications. The decrease in OD was measured at 340 nm.

All enzyme activities are expressed as milliunits per milligram of soluble protein (specific activity). One unit of enzyme activity was defined as the amount of enzyme required to transform 1 μmol of substrate per min under the above assay conditions. Soluble protein concentration was determined using the method of Bradford (1976) [49], with bovine serum albumin used as a standard.

### 4.4. Statistical Analysis

Results are expressed as the mean ± standard error of the mean (SEM). The effects of the different treatments on all the parameters studied were analyzed using two-way analysis of variance (two-way ANOVA), followed by Tukey’s HSD test. When interaction between the factors analyzed was observed, one-way ANOVA was performed for each factor independently, followed by Tukey’s HSD test. The *n* is the number of independent experiments. The confidence interval was 95%, and the differences were considered significant for values of *p* < 0.05. The statistical treatment of the data was carried out using the IBM SPSS Statistics program (IBM^®^ Armonk, NY, USA), software (version 25).

## 5. Conclusions

Induced tendinopathy (DC group) in rats produces alterations in the liver intermediary metabolism, highlighting an increase in NADPH production by the ME activity and decreased lipogenesis in the fibroblast remodeling phase (Phase II). In carbohydrate metabolism, MA has no potentiating effect, and it even decreases PK and FBPase activity; HT also did not cause any notable effect; and AA treatment produces a decrease in this metabolism. Regarding lipid metabolism, MA causes a decrease in lipogenesis at the beginning and at the end of tendinopathy and increases fatty acid oxidation during Phase I–II; HT increases lipogenesis during the inflammatory phase (Phase I); and glycine and aspartate decrease lipogenesis in general and lipolysis during the fibroblastic regeneration phase. In protein metabolism, MA treatment increases GDH and AST activity during Phase I–II; HT decreases ALT activity during the first two phases; and the AA treatment does not cause any alteration.

The results obtained show a modification of intermediary metabolism when subjects with induced tendinopathy were treated with nutritional factors implemented in their diet. Therefore, nutritional factors as a dietary supplement could be an alternative to the current invasive and ineffective treatment of tendinopathies.

## Figures and Tables

**Table 1:** *p* values obtained from two-way ANOVA analysis performed for diet and phases of tendinopathy.

	*p* Value		
Enzymes	Diet	Phases	Diet × Phases
CS	*p* < 0.01	*p* < 0.01	*p* < 0.05
ME	*p* < 0.001	*p* < 0.001	*p* < 0.001
G6PDH	*p* < 0.001	ns	*p* < 0.001
LDH	ns	*p* < 0.001	*p* < 0.05
FAS	*p* < 0.01	*p* < 0.01	*p* < 0.001
FBPase	*p* < 0.05	*p* < 0.001	*p* < 0.001
GDH	*p* < 0.05	*p* < 0.001	*p* < 0.001
AST	*p* < 0.05	*p* < 0.001	*p* < 0.001
ALT	ns	*p* < 0.001	*p* < 0.001
T-HK	ns	*p* < 0.001	*p* < 0.001
HOAD	ns	*p* < 0.001	*p* < 0.001
PK	*p* < 0.001	*p* < 0.001	*p* < 0.001

ns: No significant differences (*p* > 0.05). The enzymes: Citrate Synthase (CS), Glucose-6-Phosphate Dehydrogenase (G6PDH), Lactate Dehydrogenase (LDH), Fructose Bisphosphatase (FBPase), Total Hexokinase (T-HK), Pyruvate Synthase (PK), Malic Enzyme (ME), Fatty Acid Synthase (FAS), Hydroxyacyl-CoA Dehydrogenase (HOAD), Glutamate Dehydrogenase (GDH), Aspartate Aminotransferase (AST), and Alanine Aminotransferase (ALT).

**Table 2:** Weight monitoring.

	Initial Weight (g)	Final Weight (g)	Weight Gain (g/Kg/day)	Intake g/Kg/day
C	316.8 ± 3.3	470.0 ± 24.4	11.8 ± 0.8	73.8 ± 1.2
DC	317.7 ± 3.3	483.0 ± 7.2	10.2 ± 0.9	72.8 ± 0.9
HT	320.9 ± 3.8	487.2 ± 12.6	10.7 ± 0.8	76.2 ± 0.5
MA	321.4 ± 3.3	451.7 ± 12.0	10.8 ± 0.5	73.9 ± 0.9
AA	318.8 ± 2.8	449.5 ± 9.0	10.7 ± 0.9	74.0 ± 1.4

Average weight of rats in each of the groups: Healthy Control (C), Diseased Control (DC), Hydroxytyrosol (HT), Maslinic Acid (MA), AA: Amino Acids Glycine and Aspartate (Gly + Asp). Values are presented as mean ± SEM (*n* = 4) and were considered significantly different at *p* < 0.05.

**Table 3:** Phase I. Effect of different nutritional treatments on the enzyme activities in the liver of rats with or without TP.

Treatment	C	DC	HT	MA	AA (Gly + Asp)
Enzymes					
**CS**	6.41 ± 0.59	8.08 ± 0.63	8.51 ± 1.41	4.32 ± 0.49 ^a^	7.35 ± 1.29 ^ab^
**G6PDH**	27.5 ± 2.4 ^bAB^	32.5 ± 2.7 ^bB^	20.5 ± 3.4 ^aA^	22.4 ± 0.7 ^A^	19.0 ± 1.2 ^abA^
**LDH**	3417.5 ±92.5 ^b^	3366.6 ± 197.4 ^ab^	3339.8 ± 189.5 ^ab^	3163.3 ±52.3	3886.0 ± 271.2 ^b^
**FBPase**	34.42 ± 1.43 ^AB^	40.88 ± 1.89 ^B^	32.91 ± 1.95 ^aAB^	28.93 ± 2.64 ^aA^	40.12 ± 3.43 ^abB^
**T-HK**	1.69 ± 0.13 ^b^	1.93 ± 0.48	2.24 ± 0.19	1.77 ± 0.29 ^ab^	2.08 ± 0.26 ^ab^
**PK**	229.1 ± 10.4 ^AB^	317.3 ± 13.5 ^bC^	294.2 ± 23.1 ^BC^	187.9 ± 13.2 ^aA^	222.4 ± 12.9 ^A^
**ME**	4.86 ± 0.45 ^abA^	7.05 ± 0.77 ^B^	8.93 ± 0.17 ^cB^	3.44 ± 0.15 ^aA^	4.52 ± 0.29 ^bA^
**FAS**	0.82 ± 0.02 ^aAB^	1.13 ± 0.05 ^BC^	1.89 ± 0.04 ^D^	0.70 ± 0.04 ^aA^	1.44 ± 0.14 ^bcC^
**HOAD**	145.04 ± 10.85 ^a^	171.0 ± 12.71	218.6 ± 32.08	142.3 ± 2.48 ^a^	188.4 ± 18.05 ^ab^
**GDH**	530.1 ± 16.73 ^a^	645.4 ± 85.49	573.1 ± 0.53	567.6 ± 20.6 ^a^	606.2 ± 81.83
**AST**	702.8 ± 51.85	743.1 ± 79.36	630.5 ± 35.42	662.8 ± 52.12 ^ab^	754.7 ± 56.28 ^ab^
**ALT**	91.16 ± 12.54 ^AB^	132.2 ± 4.81 ^BC^	78.67 ± 3.43 ^aA^	89.54 ± 13.94 ^aAB^	151.7 ± 15.51 ^C^

Effect of different nutritional treatments and stages of tendinopathy on the activity (nmol/min/mg protein) of enzymes Citrate Synthase (CS), Glucose-6-Phosphate Dehydrogenase (G6PDH), Lactate Dehydrogenase (LDH), Fructose Bisphosphatase (FBPase), Total Hexokinase (T-HK), Pyruvate Synthase (PK), Malic Enzyme (ME), Fatty Acid Synthase (FAS), Hydroxyacyl-CoA Dehydrogenase (HOAD), Glutamate Dehydrogenase (GDH), Aspartate Aminotransferase (AST), and Alanine Aminotransferase (ALT) in rat liver samples with and without induced tendinopathy. Healthy Control (C), Diseased Control (DC), Hydroxytyrosol (HT), Maslinic Acid (MA), Amino Acids Glycine and Aspartate (AA (Gly + Asp)). Values are presented as mean ± SEM (*n* = 4) and were considered significantly different at *p* < 0.05. Lower case letters indicate significant differences between tendinopathy stages within each of the experimental treatments. Uppercase letters indicate significant differences between treatments within each of the tendinopathy phases. The blue color represents enzymes involved in carbohydrate metabolism, the green color represents enzymes involved in lipid metabolism, and the orange color represents enzymes involved in protein metabolism.

**Table 4:** Phase I–II. Effect of different nutritional treatments on the enzyme activities in the liver of rats with or without TP.

Treatment	C	DC	HT	MA	AA (Gly + Asp)
Enzymes					
**CS**	5.70 ± 0.51 ^A^	9.58 ± 1.23 ^B^	8.32 ± 0.24 ^AB^	7.14 ± 0.48 ^bAB^	8.75 ± 0.81 ^bAB^
**G6PDH**	26.7 ± 2.5 ^b^	21.4 ± 1.9 ^a^	23.5 ± 1.6 ^ab^	25.1 ± 4.0	19.8 ± 0.9 ^b^
**LDH**	3290.0 ± 184.1 ^b^	4079.4 ± 247.0 ^b^	3716.8 ± 222.1 ^bc^	3453.7 ± 298.4	4112.2 ± 104.3 ^b^
**FBPase**	41.06 ± 5.64 ^AB^	39.15 ± 1.39 ^AB^	42.97 ± 3.12 ^aAB^	53.07 ± 1.61 ^B^	37.81 ± 3.27 ^abA^
**T-HK**	2.58 ± 0.29 ^c^	1.81 ± 0.04	1.5 ± 0.27	1.21 ± 0.21 ^a^	2.73 ± 0.73 ^b^
**PK**	244.3 ± 38.8	241.8 ± 12.3 ^a^	344.9 ± 32.1	316.9 ± 57.6 ^ab^	272.2 ± 21.7
**ME**	6.19 ± 0.71 ^b^	6.52 ± 0.68	6.91 ± 0.32 ^b^	6.33 ± 0.79 ^b^	6.20 ± 0.41 ^c^
**FAS**	1.30 ± 0.12 ^abAB^	1.86 ± 0.17 ^BC^	1.37 ± 0.085 ^AB^	2.04 ± 0.22 ^bAB^	1.08 ± 0.06 ^abA^
**HOAD**	189.9 ± 19.6 ^abA^	184.9 ± 11.2 ^A^	202.8 ± 16.6 ^A^	321.4 ± 30.2 ^bB^	213.2 ± 22.7 ^bA^
**GDH**	683.82 ± 79.6 ^abA^	866.4 ± 88.7 ^AB^	667.1 ± 18.57 ^A^	1200.3 ± 85.5 ^bB^	668.9 ± 88.7 ^A^
**AST**	979.0 ± 116.2 ^A^	1065.6 ± 106.7 ^A^	1035.3 ± 105.7 ^A^	1513.6 ± 72.8 ^cB^	749.9 ± 70.8 ^abA^
**ALT**	196.3 ± 55.9	105.3 ± 11.2	155.7 ± 16.2 ^b^	144.4 ± 13.9 ^bc^	158.1 ± 12.7

Effect of different nutritional treatments and stages of tendinopathy on the activity (nmol/min/mg protein) of enzymes Citrate Synthase (CS), Glucose-6-Phosphate Dehydrogenase (G6PDH), Lactate Dehydrogenase (LDH), Fructose Bisphosphatase (FBPase), Total Hexokinase (T-HK), Pyruvate Synthase (PK), Malic Enzyme (ME), Fatty Acid Synthase (FAS), Hydroxyacyl-CoA Dehydrogenase (HOAD), Glutamate Dehydrogenase (GDH), Aspartate Aminotransferase (AST), and Alanine Aminotransferase (ALT) in rat liver samples with and without induced tendinopathy. Healthy Control (C), Diseased Control (DC), Hydroxytyrosol (HT), Maslinic Acid (MA), Amino Acids Glycine and Aspartate (AA (Gly + Asp)). Values are presented as mean ± SEM (*n* = 4) and were considered significantly different at *p* < 0.05. Lower case letters indicate significant differences between tendinopathy stages within each of the experimental treatments. Uppercase letters indicate significant differences between treatments within each of the tendinopathy phases. The blue color represents enzymes involved in carbohydrate metabolism, the green color represents enzymes involved in lipid metabolism, and the orange color represents enzymes involved in protein metabolism.

**Table 5:** Phase II. Effect of different nutritional treatments on the enzyme activities in the liver of rats with or without TP.

Treatment	C	DC	HT	MA	AA (Gly + Asp)
Enzymes					
**CS**	7.84 ± 0.98 ^B^	7.25 ± 0.60 ^B^	6.39 ± 0.63 ^AB^	5.05 ± 0.74 ^abAB^	4.08 ± 0.25 ^aA^
**G6PDH**	25.6 ± 1.0 ^b^	26.4 ± 3.6 ^ab^	24.5 ± 4.9 ^ab^	17.3 ± 0.3	19.6 ± 3.2 ^b^
**LDH**	2307.5 ± 156.8 ^a^	2875.4 ± 397.0 ^a^	2730.5 ± 216.8 ^a^	3008.3 ± 150.5	2757.4 ± 318.4 ^a^
**FBPase**	32.62 ± 2.46 ^AB^	43.73 ± 3.93 ^B^	31.23 ± 3.25 ^aAB^	27.31 ± 2.70 ^aA^	29.86 ± 1.86 ^aA^
**T-HK**	0.67 ± 0.04 ^aAB^	1.32 ± 0.12 ^C^	1.99 ± 0.19 ^C^	1.10 ± 0.04 ^aAB^	0.61 ± 0.03 ^aA^
**PK**	225.5 ± 8.6 ^ABC^	268.7± 12.6 ^abBC^	273.6 ± 24.0 ^C^	172.8 ± 10.8 ^aA^	207.8 ± 12.0 ^AB^
**ME**	2.65 ± 0.03 ^aA^	4.45 ± 0.44 ^B^	3.96 ± 0.40 ^aAB^	3.47 ± 0.28 ^aAB^	2.94 ± 0.28 ^aA^
**FAS**	2.00 ± 0.17 ^cB^	1.01 ± 0.06 ^A^	1.15 ± 0.07 ^A^	0.85 ± 0.02 ^aA^	0.78 ± 0.03 ^aA^
**HOAD**	228.1 ± 14.0 ^bC^	210.6 ± 17.2 ^BC^	157.5 ±10.5 ^AB^	141.7 ± 15.3 ^aA^	139.4 ± 6.7 ^aA^
**GDH**	883.4 ± 69.4 ^bB^	645.1 ± 5.95 ^A^	531.1 ± 84.4 ^A^	443.6 ± 36.7 ^aA^	499.6 ± 35.6 ^A^
**AST**	1046.5 ± 100.5 ^B^	802.5 ± 19.9 ^AB^	762.5 ± 139.6 ^AB^	529.8 ± 57.9 ^aA^	520.9 ± 56.4 ^aA^
**ALT**	125.1 ± 9.53 ^ABC^	156.4 ± 17.8 ^C^	80.38 ± 3.02 ^aA^	102.1 ± 11.2 ^abAB^	133.6 ± 13.3 ^C^

Effect of different nutritional treatments and stages of tendinopathy on the activity (nmol/min/mg protein) of enzymes Citrate Synthase (CS), Glucose-6-Phosphate Dehydrogenase (G6PDH), Lactate Dehydrogenase (LDH), Fructose Bisphosphatase (FBPase), Total Hexokinase (T-HK), Pyruvate Synthase (PK), Malic Enzyme (ME), Fatty Acid Synthase (FAS), Hydroxyacyl-CoA Dehydrogenase (HOAD), Glutamate Dehydrogenase (GDH), Aspartate Aminotransferase (AST), and Alanine Aminotransferase (ALT) in rat liver samples with and without induced tendinopathy. Healthy Control (C), Diseased Control (DC), Hydroxytyrosol (HT), Maslinic Acid (MA), Amino Acids Glycine and Aspartate (AA (Gly + Asp)). Values are presented as mean ± SEM (*n* = 4) and were considered significantly different at *p* < 0.05. Lower case letters indicate significant differences between tendinopathy stages within each of the experimental treatments. Uppercase letters indicate significant differences between treatments within each of the tendinopathy phases. The blue color represents enzymes involved in carbohydrate metabolism, the green color represents enzymes involved in lipid metabolism, and the orange color represents enzymes involved in protein metabolism.

**Table 6:** Phase III. Effect of different nutritional treatments on the enzyme activities in the liver of rats with or without TP.

Treatment	C	DC	HT	MA	AA (Gly + Asp)
Enzymes					
**CS**	5.39 ± 0.8	7.39 ± 1.74	5.60 ± 1.09	6.46 ± 0.17 ^ab^	4.75 ± 0.29 ^a^
**G6PDH**	13.1 ± 0.5 ^aAB^	18.6 ± 0.9 ^aB^	35.9 ± 1.5 ^bC^	27.1 ± 2.1 ^C^	11.8± 0.9 ^aA^
**LDH**	4031.8 ± 269.1 ^b^	4415.8 ± 245.6 ^b^	4351.5 ± 196.3 ^c^	4093.8 ± 446.4	3388.7 ± 76.0 ^ab^
**FBPase**	41.38 ± 7.60 ^A^	50.95 ± 4.73 ^AB^	58.59 ± 4.26 ^bAB^	63.46 ± 4.15 ^bB^	43.36 ± 2.92 ^bAB^
**T-HK**	2.27 ± 0.22 ^bc^	1.94 ± 0.22	1.63 ± 0.13	2.22± 0.18 ^b^	2.16 ± 0.16 ^ab^
**PK**	327.6 ± 40.6 ^B^	390.4 ± 24.4 ^cB^	341.3 ± 11.8 ^B^	412.0 ± 33.8 ^bB^	197.2 ± 28.5 ^A^
**ME**	3.97 ± 0.65 ^abA^	5.57 ± 0.82 ^AB^	8.01 ± 0.57 ^bcB^	7.60 ± 0.56 ^bB^	4.07 ± 0.33 ^abA^
**FAS**	1.44 ± 0.11 ^bAB^	2.12 ± 0.56 ^B^	1.41 ± 0.37 ^AB^	0.74 ± 0.06 ^aA^	1.57 ± 0.16 ^aAB^
**HOAD**	177.8 ± 10.3 ^abA^	204.5 ± 15.0 ^AB^	238.3 ± 12.0 ^B^	200.6 ± 19.0 ^aAB^	206.1 ± 7.1 ^bAB^
**GDH**	726.8 ± 87.2 ^ab^	792.5 ± 95.6	655.3 ± 59.6	587.6 ± 55.5 ^a^	725.7 ± 58.0
**AST**	973.3 ± 88.9	1073.3 ± 84.1	972.3 ± 90.4	787.1 ± 54.7 ^b^	927.7 ± 57.5 ^b^
**ALT**	115.4 ± 17.2 ^A^	112.0 ± 11.7 ^A^	216.1 ± 17.5 ^cB^	196.3 ± 11.3 ^cB^	160.4 ± 25.9 ^B^

Effect of different nutritional treatments and stages of tendinopathy on the activity (nmol/min/mg protein) of enzymes Citrate Synthase (CS), Glucose-6-Phosphate Dehydrogenase (G6PDH), Lactate Dehydrogenase (LDH), Fructose Bisphosphatase (FBPase), Total Hexokinase (T-HK), Pyruvate Synthase (PK), Malic Enzyme (ME), Fatty Acid Synthase (FAS), Hydroxyacyl-CoA Dehydrogenase (HOAD), Glutamate Dehydrogenase (GDH), Aspartate Aminotransferase (AST), and Alanine Aminotransferase (ALT) in rat liver samples with and without induced tendinopathy. Healthy Control (C), Diseased Control (DC), Hydroxytyrosol (HT), Maslinic Acid (MA), Amino Acids Glycine and Aspartate (AA (Gly + Asp)). Values are presented as mean ± SEM (*n* = 4) and were considered significantly different at *p* < 0.05. Lower case letters indicate significant differences between tendinopathy stages within each of the experimental treatments. Uppercase letters indicate significant differences between treatments within each of the tendinopathy phases. The blue color represents enzymes involved in carbohydrate metabolism, the green color represents enzymes involved in lipid metabolism, and the orange color represents enzymes involved in protein metabolism.

**Table 7:** Proximate analyses (% dry matter) of the experimental diets.

Proximate Analysis	Control	AA	AM	HT
Dry matter	9.48	8.15	8.11	8.74
Crude protein	14.58	17.48	13.96	14.62
Crude lipid	3.76	3.60	2.76	4.37
Ash	3.77	3.84	3.93	3.87
Nitrogen-free extract	68.41	66.93	71.24	68.40
Energetic value (KJ/100 dry weight)	1529	1547	1528	1552

Nitrogen-free extract = 100 − (crude protein + crude lipid + ash).

## Data Availability

The data presented in this study are available on request from the corresponding author.
